# Geographic Clustering of Cardiometabolic Risk Factors in Metropolitan Centres in France and Australia

**DOI:** 10.3390/ijerph13050519

**Published:** 2016-05-21

**Authors:** Catherine Paquet, Basile Chaix, Natasha J. Howard, Neil T. Coffee, Robert J. Adams, Anne W. Taylor, Frédérique Thomas, Mark Daniel

**Affiliations:** 1 Centre for Population Health Research, School of Health Sciences, Sansom Institute for Health Research, University of South Australia, Adelaide SA 5001, Australia; natasha.howard@unisa.edu.au (N.J.H.); neil.coffee@unisa.edu.au (N.T.C.); mark.daniel@unisa.edu.au (M.D.); 2 Research Center of the Douglas Mental Health University Institute, Montréal, QC H4H 1R3, Canada; 3 Inserm, UMR-S 1136, Pierre Louis Institute of Epidemiology and Public Health, Nemesis Team, Paris 75012, France; basile.chaix@iplesp.upmc.fr; 4 Sorbonne Universités, UPMC Univ Paris 06, UMR-S 1136, Pierre Louis Institute of Epidemiology and Public Health, Nemesis Team, Paris 75012, France; 5 Discipline of Medicine, The University of Adelaide, Adelaide SA 5001, Australia; robert.adams@adelaide.edu.au (R.J.A.); anne.taylor@adelaide.edu.au (A.W.T.); 6 Centre d’Investigations Préventives et Cliniques, Paris 75116, France; Thomas@ipc.asso.fr; 7 Department of Medicine, The University of Melbourne, St Vincent’s Hospital, Melbourne, Fitzroy VIC 3065, Australia

**Keywords:** Intra-Class Correlation, cross-country comparison, geographic clustering, geographic variation, cardiometabolic risk factors

## Abstract

Understanding how health outcomes are spatially distributed represents a first step in investigating the scale and nature of environmental influences on health and has important implications for statistical power and analytic efficiency. Using Australian and French cohort data, this study aimed to describe and compare the extent of geographic variation, and the implications for analytic efficiency, across geographic units, countries and a range of cardiometabolic parameters (Body Mass Index (BMI) waist circumference, blood pressure, resting heart rate, triglycerides, cholesterol, glucose, HbA_1c_). Geographic clustering was assessed using Intra-Class Correlation (ICC) coefficients in biomedical cohorts from Adelaide (Australia, *n* = 3893) and Paris (France, *n* = 6430) for eight geographic administrative units. The median ICC was 0.01 suggesting 1% of risk factor variance attributable to variation between geographic units. Clustering differed by cardiometabolic parameters, administrative units and countries and was greatest for BMI and resting heart rate in the French sample, HbA_1c_ in the Australian sample, and for smaller geographic units. Analytic inefficiency due to clustering was greatest for geographic units in which participants were nested in fewer, larger geographic units. Differences observed in geographic clustering across risk factors have implications for choice of geographic unit in sampling and analysis, and highlight potential cross-country differences in the distribution, or role, of environmental features related to cardiometabolic health.

## 1. Introduction

There has been a proliferation of place (or neighbourhood) and health research over the last two decades in which a range of attributes related to place of residence and their potential link to behavioural and clinical outcomes have been investigated in multiple contexts. Methodological heterogeneity across studies, however, precludes the assessment of robustness of these associations and their comparison across study sites [1]. For this reason replication studies have been recommended. Beyond the ascertainment of general conclusions relating to the effect of attributes of where people live on health, there is also the need to enhance our understanding of the processes which both shape and explain these observations [2]. Causal inference may be augmented through studies that seek to capitalise on “natural” variations in contextual conditions, which exist across regions and countries [3,4]. Such comparisons are starting to emerge [5,6,7]. Critical to the success of these comparisons is not only the development of comparable attributes of place and clinical outcome measures, but also an *a priori* understanding of the geographic clustering of health outcomes within differently scaled expressions of a given context as well as altogether different contexts. Gaining insights into the scale and nature of environmental influences on cardiometabolic diseases in different contexts can therefore help guide the selection of an appropriate geographic unit to be used for comparison purposes. Researchers undertaking cross-context comparisons appear to have an appreciation of measurement specification, but limited attention seems to have been paid to geographic clustering thus far.

Geographic clustering of outcomes can be assessed using either geospatial measures, such as the Moran’s I [8], or the standard Intra-Class Correlation (ICC) coefficient, each readily calculated using standard geographic administrative units and relevant to the multi-level analytic models often used in place and health studies. The ICC provides information on the degree of “relatedness” of aggregates of people in the same geographic (or social) unit, reflecting shared experiences or conditions [9]. This information is useful to orient future research on the geographic drivers of cardiovascular diseases (CVD) by analysing CVD risk factors that have the largest amount of within-area correlation and between-area variability. Information on the strength of clustering of risk factors is important to public health authorities to assist the targeting and analysis of interventions to reduce regional disparities in disease [10].

It is also known that naively analysing clustered observations as if they were independent results in underestimation of the standard error of the estimated cluster-level effect and thus the inflation of Type I error rates [11]. The ICC quantifies the bias created by not accounting for the extent of non-independence in analyses and has therefore utility in sample size calculations and accurate estimates of parameter effects and their variability in clustered designs [12]. Intra-Class Correlation is a function of the similarity of individuals within groups, relative to the similarity between groups. Thus, the extra variation, attributable to the geographic unit, is given by the ICC indexing the geographic clustering of individuals for the geographic unit being analysed. The effect of clustering depends, however, on both the cluster size and the ICC by which the ‘design effect’ can be expressed as a multiplier of the usual variance estimate [13,14]. For a given ICC, the design effect can therefore serve to quantify the extent of analytic efficiency associated with the use of differently sized geographic units (translating into different numbers of geographic units for a given region). The design effect will be close to 1.0 for large numbers of geographic units, indicating a minimal impact on analytic efficiency. The design effect can be much greater, and analytic efficiency correspondingly reduced, for limited numbers of geographic units. Understanding the effects of clustering on analytic efficiency hence has important implications for the selection and use of different geographic units in place and health analyses.

Using Australian and French cardiometabolic risk factor data from population-based cohorts in large metropolitan centres in each nation, this study of the geographic clustering of risk factors aimed to provide knowledge relevant to decisions on choice of geographic unit and interpretation of analyses of relationships between environmental factors and cardiometabolic outcomes for different places. The objectives were to describe and compare the extent of geographic variation, and the implications for analytic efficiency, across a range of cardiometabolic parameters for geographic units in these countries. In so doing, this report implicates cross-country differences in the distribution and/or role of environmental features that bear on cardiometabolic health. It also provides information that can assist place and health researchers in understanding the implications of geographic clustering of health outcomes related to decisions on geographic units for sampling and analysis.

## 2. Materials and Methods

### 2.1. Context and Data Sources

This study uses baseline data from cohort studies in metropolitan regions of Adelaide, South Australia and Paris, France. Both cohort and their study regions are described below.

#### 2.1.1. Australian Cohort and Study Region

**Figure 1:**
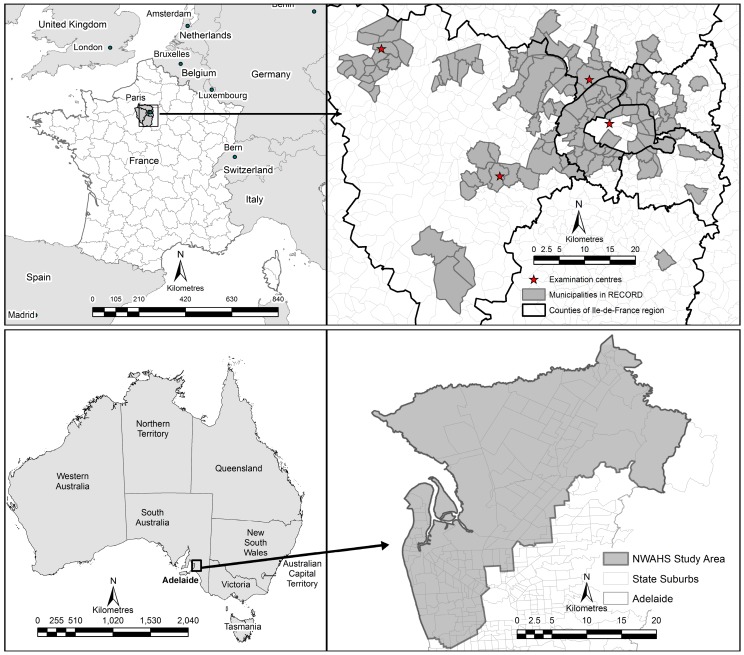
Study regions, Adelaide, South Australia and Paris, France.

Adelaide was settled in 1836 with systematic urban planning by Colonel William Light [15], featuring a square mile street grid and parklands region surrounding a central business district. The Adelaide metropolitan region has developed over time to stretch 80 km north to south, and 30 km east to west, and also to reflect a diverse ethnic and socioeconomic profile. In 2001, population density for the Adelaide Statistical Division was 587.8 persons per square kilometre. The Adelaide baseline cohort study region includes the northern and western regions of metropolitan Adelaide (see Figure 1), accounting for 38% of the city population and 28% of the population of the state of South Australia [16]. The population of the study region reflects the post-World War II migration of peoples from Europe and the United Kingdom. Relative to the state, the study region has high proportions of low income families and low full-time secondary school participation rates [17]. Despite this profile, the region covers a diverse spectrum of both high and low socioeconomic status and ethnic background residents, as well as, commercial, educational and industrial sites.

The Australian study is part of the Place and Metabolic Syndrome (PAMS) project which links geospatial data with clinical data, by which to analyse environmental attributes hypothesised to affect cardiometabolic risk and disease. Clinical data are derived from a population-based biomedical cohort, the North West Adelaide Health Study (NWAHS). Spatially-referenced data on cohort participants’ social and built environments are extracted from a comprehensive geographic information system (GIS). The NWAHS is a collaboration between the Central Northern Area Health Service (CNAHS), South Australian Department for Health and Ageing, The University of Adelaide, University of South Australia and SA Pathology [18].

The NWAHS is comprised of adults aged 18 years and over from the north-west region of Adelaide, randomly selected from the Electronic White Pages (EWP) telephone directory. To date, there have been three waves of data collection, Wave 1 (2000–2003), Wave 2 (2004–2006) and Wave 3 (2008–2010). At baseline recruitment there were 8213 eligible individuals, of which 5850 completed a Computer Assisted Telephone Interview (CATI) including socio-demographic, behavioural and self-reported health questions. These participants were invited to undertake a clinical assessment, taking approximately 45 min and conducted by trained clinical staff. Clinical measures included weight, height, waist and hip girths, blood pressure, and a fasting blood sample from which glycaemic and lipid variables were determined. These assessments conducted at either of two hospitals within the north-west region were completed by 4056 participants or 49.4% of the eligible sample, and 69.4% of those who completed the CATI. All participants provided informed consent. Relative to the study region population, cohort participants were more likely to be female, aged 45 years and over, and hold a university degree.

NWAHS participants were assigned a geo-reference that represented their residential address at all stages of data collection. Of the original baseline sample, 4041 participants had a valid geo-reference. Missing geo-references reflect incomplete residential street addresses that were not traceable following a crosscheck of paper records or telephone contact. Ethics review board approval for the PAMS project was provided by the University of South Australia (P029-10; P030-10), the Central Northern Area Health Service (HREC No. 2010010), and South Australian Department for Health and Ageing (HREC/13/SAH/57).

#### 2.1.2. French Cohort and Study Region

The city of Paris was remodelled by Baron Georges-Eugène Haussmann during the 1850s, incorporating regulations on building ways, public parks, and facilities. The grid plan of Paris saw frequent intersections and orthogonal geometry to accommodate daily population movement. According to the National Institute of Statistics and Economic Studies (Insee), the overall population density of the City of Paris in 2009 was 21,196 residents per square kilometre. Older cities such as this grew organically but to include regions less reminiscent of the original urban design. The Paris cohort study region, situated within the Île-de-France region, includes 10 of the 20 districts of Paris and 111 other municipalities of the region (see Figure 1). These municipalities are located in close proximity to Paris, in the first crown of counties around Paris and further away in the second crown of counties in the Île-de-France region. The Île-de-France region has, overall, the highest socioeconomic status in France yet also the greatest socio-territorial inequalities. These inequalities were reflected in the municipalities sampled in the French cohort. Figure 1 provides the geographic setting of the two study regions.

The RECORD Cohort Study (“Residential Environment and CORonary heart Disease” [19]) includes 7290 participants recruited between March 2007 and February 2008 [20,21]. Additional participants were recruited for a second wave of data collection (2011–2013). The study is a collaboration between UMR-S 707 (Inserm—Université Pierre et Marie Curie) and the Centre d’Investigations Préventives et Cliniques of Paris. Participants benefit from a free health check-up, offered every five years to all working and retired employees and their families by the French National Health Insurance System for Salaried Workers. Participants were recruited without *a priori* sampling during these two-hour long preventive health check-ups conducted by the Centre d’Investigations Préventives et Cliniques [22,23] in four of its health centres, located in the Paris metropolitan area (Paris, Argenteuil, Trappes, and Mantes-la-Jolie). Eligibility criteria were age 30 to 79 years, ability to complete the study questionnaires in French, and residence within the study region. Among those attending health centres and who were eligible based on age and place of residence, 10.9% were not admitted given linguistic or cognitive difficulties in completing questionnaires. Individuals admitted for participation received further information about the study from trained survey staff. Of these, 83.6% agreed to participate and completed the data collection protocol. Relative to the population attending the preventive health checks, those individuals admitted for participation in the study had greater education, and resided closer to the examination centres and in more affluent and/or low building density neighbourhoods [24].

Participants were geocoded using their residential address in 2007–2008. Research assistants corrected all incorrect or incomplete addresses with the participants by telephone. Extensive investigations with local Departments of Urban Planning were conducted to complete the geocoding. Precise spatial co-ordinates and block group codes were identified for 100% of the participants. The study protocol was approved by the French Data Protection Authority (Authorisation #907011). All participants signed an informed consent to enter the study.

### 2.2. Outcome Variables

The French and Australian studies both focused on cardiovascular health and collected information on recognised risk markers for cardiovascular diseases. The following risk markers measured at baseline were therefore used for calculation and comparison of ICCs: Body Mass Index (BMI), waist girth, systolic blood pressure, diastolic blood pressure, resting heart rate, and fasting level of triglycerides, total cholesterol, high-density lipoprotein cholesterol (HDL-C), blood glucose, and glycosylated haemoglobin (HbA_1c_). Resting heart rate was not measured in the Australian cohort, and HbA_1c_ was not measured in the French Cohort. Participants with a missing value for any outcome measure were excluded, resulting in a final sample of 6430 French and 3893 Australian participants (88.2%, and 96.3% of the original cohorts, respectively).

Both studies measured height via wall-mounted stadiometre and body mass using calibrated scales [22,25]. BMI was computed as body mass (kg)/height (m^2^). Waist girth was measured with the participant standing evenly on both feet to the nearest millimetre using an inelastic tape placed midway between the lower ribs and iliac crests on the mid-axillary line [23].

Supine brachial blood pressure was measured three times in the right arm after a 10 min rest period, using a manual sphygmomanometer [22,25]. Cuff size was selected based on arm girth. The first and the fifth Korotkoff phases were used to define systolic blood pressure (mmHg) and diastolic blood pressure (mmHg), respectively. The mean of the second and third reading of each measure was taken as the ‘true’ value [23]. For the French cohort only, resting heart rate in beats per minute was measured by electrocardiogram using a Cardionics CardioPlug device following a 5–7 min rest period [22]. 

For the Australian cohort, concentrations of fasting serum total cholesterol, HDL-C, and triglycerides, and fasting plasma glucose were measured using Chemistry Immuno Analyzer systems (Olympus AU5402 (total cholesterol/HDL-C), AU5401 (Triglycerides), Olympus AU5400 (Glucose); Olympus Optical Co. Ltd., Tokyo, Japan). Whole blood was used for HbA_1c_. In the French cohort, biomarker concentrations were measured on serum under fasting conditions (enzymatic method, automat HITACHI 917, Hitachi, Meylan, France). The HDL cholesterol was measured by direct enzymatic assay with cyclodextrin. Further detail on methodologies for both cohort studies has been provided elsewhere [26,27].

### 2.3. Administrative Geographic Units

Cohort participants were located within a hierarchy of administrative geographic units according to country-specific standards. For Adelaide, administrative geographic units compliant with 2001 Australian Standard Geographical Classification (ASGC) [28] and Census Geographic Areas [29] included, from smallest to largest: Census Collection District (CD); Statistical Local Area (SLA); State Suburb; Postal Areas (POA); and Local Government Areas (LGA). The CD, the smallest geographic unit in the ASGC, averages 220 dwellings in urban areas [28]. The four larger Australian geographic units were formed by aggregating CDs without omission or overlap. The Statistical Local Area (SLA) is a general purpose geographic unit and the base geographic unit by which population statistics other than Censuses are collected and disseminated [28]. A Postal Area (POA) is created by allocating whole CDs to approximate to Australia Post^®^ postcode areas, and the State Suburb is formed by allocating CDs to form a unit that aligns with the most recent gazetted suburb at the time of Census [29]. The Australia Post^®^ postcode and gazetted suburb are not a part of the ASGC or Census Geographic Areas and have no associated demography, and therefore, do not support direct comparisons.

For Paris, administrative geographic units included, smallest to largest: IRIS (Ilôts Regroupés pour l’Information Statistique) neighbourhoods (*i.e.*, census block groups); TRIRIS neighbourhoods (*i.e.*, census tracts); and municipalities. A further category, census blocks, refer to the portions of territory that are delimited by the street network. Given that the French participants were spread over a large territory, the number of participants per census street blocks is very low, thus this classification was not considered in the present study. IRIS neighbourhoods are defined by Insee (National Institute of Statistics and Economic Studies) based on the 1999 French Census to group census blocks largely homogeneous in socioeconomic status (SES) and housing features. IRIS neighbourhoods comprise an average of 2000 residents. TRIRIS areas are defined by Insee by grouping an average of three contiguous IRIS neighbourhoods from the same municipality. Municipalities can pertain to large, medium-size, or small towns and are the smallest administrative level for the election of representatives. The 10 districts of Paris assessed in this study were analysed as akin to the 111 independent municipalities to be included (hence “municipalities” number 121 in total). Some of the smallest municipalities in the sample were not subdivided into IRIS/TRIRIS neighbourhoods.

### 2.4. Demographic Characteristics

For each cohort, each participant’s date of birth and gender were recorded at baseline, with age being calculated from date of birth to clinic appointment. Educational attainment and income were also collected. These measures were used to describe the samples only.

### 2.5. Statistical Analyses

Two sample *t*-tests and two-sided chi-square tests were used to compare continuous and categorical demographic and clinical measures, between Australian and French samples.

ICCs were computed for each outcome variable and for each administrative geographic unit from within- and between-unit variance parameters estimated from a two-level random intercept multi-level linear model with no predictors [12]. In linear models, the ICC quantifies the strength of association for measures of individuals within the same cluster, and the proportion of the total variability attributable to between-cluster variations [30]. Thus, for continuous variables the ICC is expressed as:

(1) $$ICC=\frac{\sigma_{c}^{2}}{\sigma_{c}^{2}+\sigma^{2}}$$

where $\sigma_{c}^{2}$ is the variance in the true mean level of the outcome variable between clusters (the geographic unit variance component or between-cluster variance) and $\sigma^{2}$ is the variance in the outcome variable among study participants within a geographic unit (the individual-level variance component or within-cluster variance). The formulation based on correlated observations therefore is closely related to that based on variance components, as the ICC can be seen as a measure of the relative sizes of the two variance components. The ICC reflects the extra variation caused by the natural differences among the individuals within each cluster; it indexes dependence among individuals within a given cluster. Models were estimated with SAS software (version 9.1, SAS Institute Inc., Cary, NC, USA) using the Mixed procedure.

The design effect can be calculated as the ratio of the variance of the estimate under the actual (clustered) design to the variance of the estimate obtained assuming the same data to have come from a simple random sample [13]. It quantifies the analytic efficiency of clustered designs and is used in sample size and statistical power calculations, and to adjust statistics naively generated under the assumption of independence. In this study, design effects were derived from ICC estimates by the following equation:

(2) $$\text{Design Effect}=1+\left(\tilde{n}-1\right)\times \mathrm{ICC}$$

where $\tilde{n}$ is the harmonic mean number of participants per unit for each administrative geographic unit. The harmonic mean was used due to sample sizes (participant numbers within unit) being unequal within geographic units of a given type. It provides an estimate of the equivalent sample size for each group under a balanced design that takes into account the loss of statistical power attributed to the unequal sample sizes.

ICCs were pooled across outcomes and geographic units using meta-analytic methods for correlations using the MedCalc Statistical Software version 16.2.1 (MedCalc Software bvba, Ostend, Belgium [31]).

## 3. Results

### 3.1. Descriptive Statistics

Table 1 provides descriptive statistics for the eight administrative geographic units analysed, five Australian and three French, expressing the number of geographic units within the study regions, the median population accounted for by each geographic unit, median size (area) of geographic units, and median number of participants per geographic unit. Within each geographic unit, there was variation in the number of participants per unit, justifying the use of the harmonic mean for calculating design effects. In terms of median population count, the Australian State Suburb was comparable to the French IRIS, and the Australian POA aligned most closely to the French TRIRIS. When considering units of comparable sizes based on area, French participants were spread over a larger number of geographic units and had fewer participants per geographic units.

Table 2 provides descriptive statistics for cohort participants in each country. French participants were very slightly younger and included proportionately more men than did the Australian sample. The French had lower BMIs, smaller waists, and lower diastolic blood pressure and triglycerides level but higher total cholesterol, HDL-cholesterol and fasting glucose levels. Descriptive information related to educational attainment and household income is given but not compared due to the lack of comparability between these measures across the two samples.

### 3.2. Spatial Clustering

Variance estimates, ICCs and design effects for cardiometabolic outcomes are given in Table 3 and Table 4 for the Australian and French cohorts, respectively. The median ICC was 0.01 indicating that 1% of variance in the cardiometabolic outcomes was attributable to variation between spatial units and that the correlation between measures from participants living in the same spatial unit was 0.01.

With the exception of clustering of HDL and fasting glucose at the CD level, all other Australian ICCs were under 2%, with particularly low levels of clustering obtained for diastolic blood pressure, total cholesterol and triglycerides. Within-area correlation was also found to be particularly low for diastolic blood pressure and triglycerides in the French sample. In the French sample, BMI and resting heart rate (not available in the Australian sample) showed the greatest level of clustering, with ICCs ranging from 2.39% to 4.80%, with the remaining outcomes having ICCs all below 2%. In the Australian sample, between-area variation was greatest for HbA_1c_ (not available in the French sample), with ICCs ranging from 2.87% to 4.91%. Noticeable differences across the two samples were found for total cholesterol and BMI, with clustering being greatest in the French sample.

ICCs and design effects pooled across outcome measures are reported for all spatial units in Table 5. In both samples, clustering was strongest amongst smaller spatial units whereas the opposite was found for design effects, consistent with their weighting by sample size within units. Specifically, in Australia, CDs and State Suburbs showed the greatest clustering, but this varied by type of outcome: clustering was greatest for State Suburbs for anthropometric and blood pressure measurements, and for CDs for cholesterol and glycaemic measures. In the French sample, the smallest unit (IRIS) was associated with the greatest level of clustering for most outcomes, with the exception of anthropometric measures and diastolic blood pressure.

## 4. Discussion

This study of two population-based samples in different nations indicates that the proportion of variability in cardiometabolic risk factors explained by variation between geographic administrative units was overall relatively low, as indicated by a median ICC of 1% (Table 3 and Table 4). This modest level of clustering is consistent with several previous reports concerning the geographic clustering of cardiometabolic risk [33], mortality [34], self-reported health problems, quality of life and well-being [35], yet lower than indicated by one report [36]. Our results align, however, with community-level ICCs for cardiometabolic risk factors in a six-community heart health intervention project [10].

Despite the overall low level of clustering, the degree of clustering varied according to geographic unit, risk factor, and population setting. Variations in clustering by geographic unit and health outcome are consistent with previous investigations [35]. Considering both samples together, clustering was especially noticeable for BMI, resting heart rate and HbA_1c_, but less so for blood pressure and lipidaemic outcomes. Blood pressure and lipid measures are routinely assessed in clinical settings and largely managed through pharmacological intervention. This contrasts with the management of anthropometric and glycaemic risk factors, which are largely (although not exclusively) recommended to be addressed by lifestyle change. The greater successes achieved through pharmacological approaches compared to preventive and health promotion efforts could explain why risk factors such as blood pressure and lipidaemia showed less variability than anthropometric and glycaemic outcomes. This interpretation is also consistent with the relatively higher within-area correlation observed for systolic as opposed to diastolic blood pressure in both samples. Poor blood pressure control is common among treated hypertensive patients and has been shown to be largely attributed to poor systolic blood pressure control [37]. The above interpretation may also be partly coherent with the relatively high clustering that was found for resting heart rate, as resting heart rate control is not as commonly considered a clinical target as blood pressure control [38].

More pronounced clustering (ICC > 2%) was observed for HbA_1c_ in the Australian sample, and for BMI and resting heart rate in the French sample. Design effects for these measures were consistently greater for the larger geographic administrative units in each nation. Markedly more clustering was observed for HbA_1c_ than for fasting glucose, these ICCs 4.91 and 2.69, respectively, in the Australian sample. Although both correspond to glycaemic control, HbA_1c_ is an indicator of long-term glycaemia and has been shown to respond to social environmental stress [39,40], and also to vary with race and ethnicity [41,42]. Both social stressors and ethnic composition are likely to vary locally, potentially explaining the high level of clustering found for HbA_1c_. With respect to resting heart rate, a previous study based on the French Cohort [43] showed that higher resting heart rates were associated with lower individual- and area-level socioeconomic status as well as measures of physical inactivity and larger waist girth. The fact that these factors each exhibit geographic variations in the French region examined might explain the significant level of geographic clustering that was detected.

Although the extent of clustering in waist girth did not greatly differ between samples, clustering was greater for BMI in the French sample. Median BMI was within the overweight range for the Australian sample but within the healthful range for the French sample. This finding may reflect variation in behavioural norms between the study regions. The French study has reported lesser geographic clustering for waist girth that for BMI, contrary to previous reports where more comparable clustering was observed for these anthropometric outcomes [44,45]. A *post-hoc* analysis of the data suggested that clustering in waist girth was of a magnitude comparable to that of BMI, once statistical models were adjusted for age and gender. For example, for IRIS the ICCs were 5.6% for BMI and 4.0% for waist girth following adjustments.

The ICCs in the current study displayed sizeable variability and the corresponding design effects illustrate that even low ICCs will have substantial implications for analytic efficiency, depending on the size of the geographic administrative units used in analysis. What this study contributes beyond prior reports on geographic clustering is the demonstration, consistent across two different settings, that the extent of clustering given by the ICC is inversely related to the size of the geographic administrative unit in which individuals are clustered, whereas analytic inefficiency indexed by design effects greater than 1.0 is positively related to the size of the geographic unit in which individuals are clustered.

For a given population sample in a given geographic setting, the smallest geographic administrative unit will number greatest but include the least study participants, whereas the largest geographic unit will number least but include the greatest numbers of study participants. Decisions on the most appropriate scale and corresponding geographic administrative unit to use in spatial epidemiological studies must consider therefore the methodological relationship between analytic efficiency and clustering, and *a priori* the theoretical basis for a certain scale of observation and analysis.

As shown here, analytic efficiency is improved as the number of geographic units increases, even though parallel reductions in the size of geographic units generate reductions in the median number of study participants per geographic unit. This relationship reflects the fact that statistical power varies directly with the precision by which the mean level of a clustered outcome can be estimated, given by the inverse of:

(3) $$\frac{\sigma_{c}^{2}+\frac{\sigma^{2}}{n}}{c}$$

where $\sigma_{c}^{2}$ is the variance in the true mean level of the outcome variable among clusters (the geographic unit variance component) and $\sigma^{2}$ is the variance in the outcome variable among study participants within a geographic unit (the individual-level variance component). The equation shows that if $\sigma_{c}^{2}$ is large relative to $\sigma^{2}$, then only modest gains will accrue by increasing the number (*n*) of study participants per geographic unit, whereas far larger gains in power can be obtained if the number of clusters (*c*) is increased.

Clustering estimates provide an understanding of the scale at which environmental influences operate [46] and, with estimates of the design effect and analytic efficiency, can inform the selection of administrative geographic units to be used for local as well as cross-country comparisons. Overall, greater clustering was found for smaller geographic units such as the French IRIS and the Australian Collection District and State Suburbs as opposed to larger units. Contrary to the Australian Collection District and French IRIS units, which had few participants per unit, Australian State Suburb and French TRIRIS units were found to have relatively high variability while still maximising the number of geographic units and participants per unit available for analysis and thus providing greater statistical power for analyses conducted at that level. Final decisions regarding the selection of appropriate units for cross-site comparisons need to be informed by not only the above observations but also relevant theory, and the nature and expected variability of the environmental exposure hypothesised to explain the geographic clustering in outcomes.

The study represents a first step in an international collaborative effort to investigate differences between countries in place and health relationships. Both studies have already yielded evidence that features of geographic areas do reflect local variations in cardiometabolic risk factors. For instance, the French study has documented associations between individual and neighbourhood socio-economic status (SES) and resting heart rate [43]; between neighbourhood SES, neighbourhood urbanicity, supermarket characteristics and BMI [21,44,45]; and between socioeconomic, physical, service, and social environment characteristics and blood pressure [20,47]. The Australian study has reported on associations between public open space [48] measures, a wealth indicator derived from property value [49], walkability [50], and socioeconomic conditions of areas [51,52] and a range of cardiometabolic risk markers. The next step in this Australia–France comparison is to derive common measures of these social and built environmental features, and using appropriate geographic administrative units, assess the degree to which they can explain the local-area geographic variations in cardiometabolic health illustrated by the present study.

The current study examined geographic variation simply in the prevalence of cardiometabolic risk factors. This approach precludes inference as to whether individual socioeconomic conditions or environmental conditions shaped the geographic distribution of health outcomes or whether relatively healthier (or unhealthy) individuals tended to migrate toward particular areas. Further research is needed to assess whether risk factors in participants followed up over time retain the same extent of geographic patterning. Differences and/or similarities between Adelaide and Paris also require follow-up assessment. A greater residential density of study participants in the Paris region may have contributed to the slightly higher average level of geographic clustering observed for the French sample. The French study also covered a larger area, which resulted in fewer participants within units with comparable sizes. The study assessed risk factors for chronic conditions characterised by very long induction periods. It is not known for how long participants had lived in a given geographic unit, which may have had an impact on the extent of clustering found. Finally, this study sought to identify the geographic administrative units that had the greatest utility, in terms of both clustering and analytic efficiency, across the two study contexts by which to inform cross-country comparisons. For this reason, the ICC, estimating clustering within pre-determined administrative units, was preferred over additional geographic clustering statistics (e.g., Moran’s I) that assess the clustering of discrete point locations over a given space continuum.

## 5. Conclusions

Consistent with previous studies, our findings suggest that clustering of health-related outcomes by geographic units was, overall, modest in both contexts but more pronounced for certain types of cardiometabolic risk factors. This suggests that decisions on whether cardiometabolic risk clustering is present and/or requiring further investigation and/or intervention in a given population should be based on a range of clinical risk factors. The study also indicated that the geographic clustering of cardiometabolic factors varied according to the type and size of administrative areas considered, and study setting. Across the two study settings, the extent of clustering given by the ICC was inversely related to the size of the geographic administrative unit in which individuals are clustered, but the analytic inefficiency given by design effects greater than 1.0 was related to the size of the geographic administrative unit in which individuals were clustered. These results show that decisions on appropriate scale and the corresponding geographic administrative unit to use in geo-spatial epidemiological studies require (1) an appreciation of the methodological relationship between analytic efficiency and clustering; (2) an understanding of the clustering specific to the risk factor of interest in a given setting; and (3) an *a priori* theoretical basis for the scale of observation and analysis for the given environmental exposure under investigation.

## Figures and Tables

**Table 1:** Descriptive statistics for Australian (Adelaide) and French (Paris) administrative geographic units analysed between studies.

Characteristic		Australian Geographic Units					French Geographic Units		
		CD ^2^	State Suburb	POA	SLA	LGA	IRIS	TRIRIS	Municipalities
**Number of geographic units**		767	143	46	20	6	1866	660	121
**Total population count ^1^**	Median	480	2264	6170	23,883	82,788	2432	7983	30,509
	Q1–Q3	342–629	1063–3737	2357–9622	18,525–26,957	54,442–99,688	2087–2942	6939–9169	18,705–53,577
**Size (area)**	Median (km^2^)	0.24	1.47	6.76	17.59	55.17	0.16	0.66	5.80
	Q1–Q3 (km^2^)	0.17–0.33	1.01–2.67	4.39–12.81	12.04–24.33	38.06–94.33	0.07–0.34	0.29–1.30	3.67–8.92
**Number of participants**	Median	5	21	70	214	581	3	9	35
	Q1–Q3	3–7	9–40	37–124	133–255	530–908	2–5	6–13	19–62
	Coefficient of Variation	56.46	88.08	75.09	57.63	52.61	65.72	57.47	108.48
	Arithmetic Mean	5.16	27.66	86.00	197.80	659.33	3.45	9.74	53.14
	Harmonic Mean	3.40	9.43	27.80	46.56	459.54	2.23	6.02	11.59

^1^ Source: Australian Bureau of Statistics 2001 Population and Housing Census, 2006 French population Census. ^2^ Abbreviations: CD, Census Collection District; POA, Postal Area; SLA, Statistical Local Area; LGA, Local Government Area; IRIS, Ilôts regroupés pour l’information statistique; TRIRIS: Groups of around three IRIS areas.

**Table 2:** Descriptive statistics for population-based, metropolitan area Australian and French biomedical cohort participants.

Measure		Adelaide, Australia (*n* = 3893)		Paris, France (*n* = 6430)		*p*
		Mean (SD) or *n* (%)	Median	Mean (SD) or *n* (%)	Median	
Age		50.5 (16.4)	50	49.6 (11.2)	49	0.02
Gender	Male	1851 (47.5%)		4197 (65.3%)		<0.0001
	Female	2042 (52.5%)		2233 (34.7%)		
University education ^1^	Yes	446 (11.8%)		2472 (38.4%)		-
	No	3327 (88.2%)		3905 (60.7%)		
	Missing			53 (0.8%)		
Annual Income ^2^		38,362 (26,027) AUD	35,000 AUD	19,858 (13,591) €	16,800 €	-
BMI		27.8 (5.5)	27.1	25.4 (4.2)	24.9	<0.0001
Waist girth (cm)	Male	98.4 (13.0)	97.5	88.9 (10.8)	88	<0.0001
	Female	87.1 (14.1)	85	77.7 (11.9)	76	<0.0001
Systolic blood pressure (mmHg)		128 (18.6)	126	127.5 (17.2)	126	0.17
Diastolic blood pressure (mmHg)		80.6 (10.2)	80	76.6 (10.6)	76	<0.0001
Resting heart rate		-	-	62.7 (10.2)	62	-
Triglycerides (mg/dL)		129.6 (92.3)	106.3	97.8 (50.6)	85	<0.0001
Total cholesterol (mg/dL)		202.8 (40.8)	201.1	214.7 (38.5)	214	<0.0001
HDL (mg/dL)		52.8 (14.7)	50.3	53.3 (13.6)	51	0.08
Fasting glucose (mg/dL)		94.6 (25.1)	90.1	98.4 (14.6)	97	<0.0001
HbA_1c_ (%)		5.6 (0.8)	5.5	-	-	-

^1^ Australian sample: University graduates; French sample: At least two years of university education; ^2^ Australian sample: Household income; French sample: Income per consumption unit [32], average exchange rate (1999–2006): 1 AUD = 0.60 €.

**Table 3:** Variance components, ICC and design effect for Adelaide, Australia, sample (*n* = 3893).

Outcome	Geographic Unit	Between-Cluster Variance	Within-Cluster Variance	ICC ^2^ (%)	Design Effect
**BMI**	CD	0.37	29.48	1.26	1.03
	State Suburb	0.49	29.39	1.64	1.14
	POA	0.27	29.57	0.89	1.24
	SLA	0.40	29.48	1.33	1.60
	LGA	0.30	29.55	0.99	5.55
**Waist girth**	CD	1.50	214.68	0.69	1.02
	State Suburb	2.81	213.55	1.30	1.11
	POA	2.17	213.85	1.00	1.27
	SLA	2.42	213.95	1.12	1.51
	LGA	1.70	214.57	0.79	4.61
**Diastolic blood pressure**	CD	0.07	103.66	0.07	1.00
	State Suburb	1.08	102.66	1.04	1.09
	POA	0.48	103.26	0.47	1.12
	SLA	0.64	103.16	0.62	1.28
	LGA	0.34	103.39	0.33	2.52
**Systolic blood pressure**	CD	1.38	345.96	0.40	1.01
	State Suburb	6.36	341.38	1.83	1.15
	POA	1.86	345.58	0.54	1.14
	SLA	2.25	345.29	0.65	1.29
	LGA ^1^				1.04
**HDL-C**	CD	5.35	212.14	2.46	1.06
	State Suburb	2.52	214.86	1.16	1.10
	POA	2.14	215.13	0.99	1.26
	SLA	2.67	215.06	1.23	1.56
	LGA	1.27	216.25	0.58	3.68
**Total Cholesterol**	CD	16.04	1644.79	0.97	1.02
	State Suburb	1.14	1659.67	0.07	1.01
	POA, SLA, LGA ^1^				
**Triglycerides**	CD	39.11	8477.37	0.46	1.01
	State Suburb	62.12	8454.39	0.73	1.06
	POA	59.54	8452.96	0.70	1.19
	SLA	81.17	8444.55	0.95	1.43
	LGA	39.84	8477.45	0.47	3.14
**Fasting glucose**	CD	16.96	613.10	2.69	1.06
	State Suburb	10.18	620.19	1.62	1.14
	POA	8.68	620.68	1.38	1.37
	SLA	6.76	622.76	1.07	1.49
	LGA	4.73	625.08	0.75	4.44
**HbA_1C_**	CD	0.03	0.60	4.91	1.12
	State Suburb	0.02	0.60	3.66	1.31
	POA	0.02	0.61	3.20	1.86
	SLA	0.02	0.61	2.98	2.36
	LGA	0.02	0.61	2.87	14.16

^1^ Non-convergence of the model; ^2^ ICCs were calculated on variance estimates reported with four decimal points. Abbreviations: ICC, Intra-Class Correlation; HDL-C, high-density lipoprotein cholesterol; CD, Census Collection District; POA, Postal Area; SLA, Statistical Local Area; LGA, Local Government Area.

**Table 4:** Variance components, ICC and design effect for Paris, France, sample (*n* = 6430).

Outcome	Geographic Unit	Between-Cluster Variance	Within-Cluster Variance	ICC ^2^ (%)	Design Effect
**BMI**	IRIS	0.75	16.73	4.29	1.05
	TRIRIS	0.76	16.74	4.34	1.22
	Municipalities	0.42	17.09	2.39	1.25
**Waist girth**	IRIS	1.54	151.84	1.00	1.01
	TRIRIS	2.81	150.59	1.83	1.09
	Municipalities	2.30	151.23	1.49	1.16
**Diastolic blood pressure**	IRIS	0.42	111.53	0.37	1.00
	TRIRIS	0.66	111.29	0.58	1.03
	Municipalities	1.21	110.79	1.08	1.11
**Systolic blood pressure**	IRIS	5.70	290.44	1.92	1.02
	TRIRIS	1.63	294.50	0.55	1.03
	Municipalities	2.85	293.49	0.96	1.10
**Resting heart rate**	IRIS	4.96	98.17	4.80	1.06
	TRIRIS	3.77	99.25	3.65	1.18
	Municipalities	3.23	100.02	3.12	1.33
**HDL-C**	IRIS	3.64	182.55	1.95	1.02
	TRIRIS	2.56	183.63	1.37	1.07
	Municipalities	1.51	184.69	0.81	1.09
**Total Cholesterol**	IRIS	28.77	1454.94	1.93	1.03
	TRIRIS	15.74	1467.96	1.06	1.06
	Municipalities	7.54	1476.45	0.50	1.05
**Triglycerides**	IRIS	16.61	2542.59	0.64	1.01
	TRIRIS ^1^	-	-	-	-
	Municipalities	9.21	2550.26	0.35	1.04
**Fasting glucose**	IRIS	3.45	209.44	1.62	1.02
	TRIRIS	1.32	211.58	0.62	1.03
	Municipalities ^1^	-	-	-	-

^1^ Non-convergence of the model; ^2^ ICCs were calculated on variance estimates reported with four decimal points. Abbreviations: ICC, Intra-Class Correlation; HDL-C, high-density lipoprotein cholesterol; IRIS, Ilôts regroupés pour l’information statistique; TRIRIS: Groups of around three IRIS areas.

**Table 5:** Mean Intra-Class Correlation and design effect according to geographic unit, Australia and France samples.

Country	Geographic Unit	ICC (%)		Design Effect	
		Pooled Estimate	95% CI	Mean	SD
**Australia**	CD	1.54	0.50, 2.58	1.04	0.04
	State Suburb	1.45	0.41, 2.49	1.12	0.08
	POA	1.14	0.04, 2.25	1.31	0.24
	SLA	1.24	0.14, 2.35	1.57	0.34
	LGA	0.97	−0.22, 2.15	5.44	3.97
**France**	IRIS	2.07	1.07, 3.08	1.02	0.02
	TRIRIS	1.77	0.74, 2.79	1.08	0.07
	Municipalities	1.36	0.53, 2.18	1.13	0.11

## Abbreviations

The following abbreviations are used in this manuscript:

ASGC	Australian Standard Geographical Classification
BMI	Body Mass Index
CATI	Computer Assisted Telephone Interview
CD	Census Collection District
CNAHS	Central Northern Adelaide Health Service
EWP	Electronic White Pages
HDL	high: density lipoprotein
HbA_1c_	blood glycated haemoglobin
ICC	Intra: Class Correlation
IRIS	Ilôts regroupés pour l’information statistique
IMVS	Institute of Medical and Veterinary Science
LGA	Local Government Areas
NHMRC	National Health and Medical Research Council
NWAHS	North West Adelaide Health Study
POA	Postal Areas
SES	Socio-Economic Status
SLA	Statistical Local Area
TRIRIS	Groups of around three IRIS areas
